# A Novel Oral Arginase 1/2 Inhibitor Enhances the Antitumor Effect of PD-1 Inhibition in Murine Experimental Gliomas by Altering the Immunosuppressive Environment

**DOI:** 10.3389/fonc.2021.703465

**Published:** 2021-08-24

**Authors:** Paulina Pilanc, Kamil Wojnicki, Adria-Jaume Roura, Salwador Cyranowski, Aleksandra Ellert-Miklaszewska, Natalia Ochocka, Bartłomiej Gielniewski, Marcin M. Grzybowski, Roman Błaszczyk, Paulina S. Stańczak, Paweł Dobrzański, Bozena Kaminska

**Affiliations:** ^1^Laboratory of Molecular Neurobiology, Nencki Institute of Experimental Biology of the Polish Academy of Sciences, Warsaw, Poland; ^2^Postgraduate School of Molecular Medicine, Medical University of Warsaw, Warsaw, Poland; ^3^OncoArendi Therapeutics SA, Warsaw, Poland

**Keywords:** arginase inhibitor, tumor microenvironment, glioma associated microglia and macrophages, immune checkpoint inhibitor, immunotherapy

## Abstract

Glioblastomas (GBM) are the common and aggressive primary brain tumors that are incurable by conventional therapies. Immunotherapy with immune checkpoint inhibitors is not effective in GBM patients due to the highly immunosuppressive tumor microenvironment (TME) restraining the infiltration and activation of cytotoxic T cells. Clinical and experimental studies showed the upregulation of expression of the arginase 1 and 2 (ARG1 and ARG2, respectively) in murine and human GBMs. The elevated arginase activity leads to the depletion of L-arginine, an amino-acid required for the proliferation of T lymphocytes and natural killer cells. Inhibition of ARG1/2 in the TME may unblock T cell proliferation and activate effective antitumor responses. To explore the antitumor potential of ARG1/2 inhibition, we analyzed bulk and single-cell RNA sequencing (scRNA-seq) data from human and murine gliomas. We found the upregulation of *ARG1/2* expression in GBMs, both in tumor cells and in tumor infiltrating microglia and monocytes/macrophages. We employed selective arginase inhibitors to evaluate if ARG1/2 inhibition *in vitro* and *in vivo* exerts the antitumor effects. A novel, selective ARG1/2 inhibitor - OAT-1746 blocked microglia-dependent invasion of U87-MG and LN18 glioma cells in a Matrigel invasion assay better than reference compounds, without affecting the cell viability. OAT-1746 effectively crossed the blood brain barrier in mice and increased arginine levels in the brains of GL261 glioma bearing mice. We evaluated its antitumor efficacy against GL261 intracranial gliomas as a monotherapy and in combination with the PD-1 inhibition. The oral treatment with OAT-1746 did not affect the immune composition of TME, it induced profound transcriptomic changes in CD11b^+^ cells immunosorted from tumor-bearing brains as demonstrated by RNA sequencing analyses. Treatment with OAT-1746 modified the TME resulting in reduced glioma growth and increased antitumor effects of the anti-PD-1 antibody. Our findings provide the evidence that inhibition of ARG1/2 activity in tumor cells and myeloid cells in the TME unblocks antitumor responses in myeloid cells and NK cells, and improves the efficacy of the PD-1 inhibition.

## Introduction

Glioblastoma (GBM, WHO grade IV glioma) is the most common and aggressive primary brain tumor in adults. While the available treatments may slow down the progression of GBM and reduce neurological symptoms, the disease remains incurable. The standard treatment for GBM patients is surgical resection followed by radiation and oral chemotherapy with temozolomide (TMZ). Despite improvements in imaging, surgical techniques, radiotherapy and chemotherapy, GBM inevitably recurs and the prognosis of patients with GBM remains poor, with a median overall survival of 15 months (1, 2). The ability of tumors to modify the surrounding microenvironment and evade the immune system is increasingly recognized as an important determinant of cancer progression and patient prognosis (3). GBMs are infiltrated with various myeloid cells which do not activate their proper functions but instead they are tumor supportive and create the immunosuppressive tumor microenvironment (TME), poorly infiltrated with cytotoxic T lymphocytes and natural killer (NK) cells being frequently deficient in their antitumor activity (4).

Immune checkpoint inhibitor-based therapies provided an effective strategy to enhance antitumor immune responses in many solid cancers (5). Programmed cell death 1 (PD-1, CD279), an immune checkpoint surface receptor expressed on lymphocytes, is a mediator of immune suppression in a variety of tumors, including GBM (6). Binding of PD-1 with its ligands B7-H1 (PD-L1) or B7-DC (PD-L2) induces apoptosis or exhaustion of activated immune cells. Blockade of this interaction enhances the antitumor activity of the immune system (7). While first studies of the adjuvant therapy with pembrolizumab (a monoclonal antibody against human PD-1) in GBM patients demonstrated some benefits (8), further studies showed no survival improvement (9, 10). The recent results from a randomized phase III trial CheckMate143 with nivolumab (an anti-PD-1 antibody) did not show the improved survival of patients with recurrent GBMs compared to those treated with bevacizumab, an anti-VEGF-A antibody (11). Genomic and transcriptomic analysis of GBM patients treated with anti-PD-1 antibodies revealed a significant enrichment of the immunosuppressive transcriptomic signature in non-responders, along with differences in T cell clonal diversity and tumor microenvironment profiles (12). Growing evidence suggests that the clinical response to immunotherapies is restricted by various resistance mechanisms, such as a strong immunosuppression induced by the tumor infiltrating myeloid cells (13, 14).

Glioma-associated microglia and macrophages (GAMs) accumulate in malignant gliomas and are key drivers of tumor invasion and immunosuppression. GAMs promote tumor progression and jointly with myeloid-derived suppressor cells (MDSCs) modulate antitumor immune responses in multiple ways (15).

Both malignant cells and tumor-infiltrating myeloid cells in murine and human gliomas upregulate the expression of arginase (16, 17) and the resulting changes in the L-arginine metabolism are one of the most prominent mechanisms contributing to immunosuppression (18). There are two arginase isoforms (ARG1 and ARG2), catalyzing the same biochemical reaction, but differing in subcellular localization, expression, and regulation. ARG1 is a cytosolic protein, while ARG2 is mainly localized in the mitochondria (19). Arginase catalyzes the hydrolysis of L-arginine to urea and L-ornithine, thereby depleting extracellular L-arginine (20). T cells are auxotrophic for L-arginine and require this amino acid for the rapid and successive proliferation that follows T cell receptor activation of effector cells (21). Expression of ARG1 is a defining feature of immunosuppressive myeloid cells that are highly enriched in the TME, and the role of ARG1-expressing MDSCs in altering T-cell responses in cancer patients is well established (22, 23). CB-1158, an arginase 1 inhibitor synthesized at Calithera Biosciences blocked myeloid cell-mediated suppression of T cell proliferation *in vitro* and reduced tumor growth in several mouse models of non-CNS tumors (CT26, LLC, B16, and 4T1 tumors). The ARG1 inhibitor was effective as a single agent or in combination with checkpoint blockade (anti-PD-L1), adoptive T cell and NK cell transfer, and chemotherapy with gemcitabine. The treatment with CB-1158 increased tumor-infiltrating CD8^+^ T cells and NK cells, inflammatory cytokines, and expression of several interferon-inducible genes (24). CB-1158 advanced to clinical trials for patients with non-CNS malignancies (NCT02903914).

In the present study, we provide the compelling evidence that OAT-1746, a novel and oral small-molecule inhibitor of ARG1/2, affects glioma-microglia interactions *in vitro*, accumulates in the brain and modulates the TME of murine intracranial gliomas. We demonstrate that OAT-1746 works synergistically with PD-1 blockade and improves antitumor immune responses against gliomas.

## Materials and Methods

### Glioma Cell Cultures

Human glioblastoma cell lines LN18, U87-MG (U87) (ATCC, Manassas, VA) were cultured in Dulbecco’s modified Eagle’s medium (DMEM) supplemented with 10% fetal bovine serum (FBS) (Gibco, MD, USA) and antibiotics (100 U/mL penicillin, 100 μg/mL streptomycin). GL261 tdTomato^+^luc^+^ murine glioma cells were cultured in DMEM with the addition of 10% FBS, antibiotics and 100 μg/mL G418 (Invivogen, San Diego, CA, USA). Cells were cultured in a humidified atmosphere CO_2_/air (5%/95%) at 37°C (Heraeus, Hanau, Germany).

### Microglial Cell Cultures

Mouse immortalized microglial BV2 cells were cultured in Dulbecco’s modified Eagle’s medium GlutaMAX™ (DMEM GlutaMAX™) supplemented with 2% fetal bovine serum (Gibco, MD, USA) and antibiotics (100 U/mL penicillin, 100 μg/mL streptomycin) in a humidified atmosphere CO_2_/air (5%/95%) at 37°C (Heraeus, Hanau, Germany).

Primary microglial cultures were prepared from cerebral cortices of P0–P2 C57BL/6J mice as described (25). Briefly, after stripping off the meninges and enzymatic brain dissociation the cells were collected and seeded onto the culture flasks. After 48 h, cell cultures were washed three times with phosphate-buffered saline (PBS) to remove debris. Primary cultures were kept in DMEM supplemented with 10% FBS, 2 mM L-glutamine, 100 U/mL penicillin, and 100 µg/mL streptomycin (Gibco, MD, USA). Microglia were isolated by gentle shaking for 1 h at 100 RPM at 37°C. Detached microglia were collected by centrifugation, counted, and checked for viability. Microglia cultures were used for experiments 48 h after seeding to ensure that the cells were quiescent.

### Recombinant Arginase Activity Assays

The inhibitory activity towards hARG1 and hARG2 enzymes was assessed using recombinant enzymes biosynthesized using a prokaryotic expression system (*E. coli*) and purified by fast protein liquid chromatography (FPLC). Briefly, recombinant enzymes were incubated with the tested compounds for 1 h at 37°C in the reaction buffer (100 mM sodium phosphate buffer, 130 mM NaCl, 1 mg/mL BSA, pH 7.4) containing substrate (10 mM L-arginine hydrochloride) and cofactor (200 µM MnCl_2_). The assay is based on the detection of urea, which is generated during the conversion of L-arginine into L-ornithine catalyzed by arginases. To visualize the product, we adding a mixture of reagent A (4 mM oPA, 50 mM boric acid, 1 M sulfuric acid, 0.03% Brij-35) and reagent B (4 mM NED, 50 mM boric acid, 1 M sulfuric acid, 0.03% Brij-35) in equal proportions. The absorbance was measured at 515 nm. The urea production in the absence of any tested compound was considered as maximal enzyme activity. The absorbance in the absence of arginase (background) was considered as zero activity. Two reference compounds OAT-81 (ABH, no. 222638-65-5) and OAT-90 (2-amino-6-borono-2-(2-(3-(2,4-dichlorophenyl)propyloamino)ethyl) hexanoic acid, and two novel inhibitors OAT-1617 and OAT-1746 (the last three synthesized at OncoArendi Therapeutics) were tested. Normalized values were analyzed using GraphPad Prism 7.0 software and the IC_50_ values were determined.

### Invasion Assays

BV2 cells were plated onto a 24-well plate at the density of 4×10^4^. After 24 h, the invasion assay was performed with tissue culture inserts (6.5 mm Transwell^®^ with 8.0 µm Pore Polycarbonate Membrane Insert, Corning, NY, USA) coated with the Growth Factor Reduced Matrigel™ Matrix (BD Biosciences, San Diego. CA, USA). The Matrigel™ Matrix (50 µL of 1 mg/mL stock solution diluted in fresh DMEM) was dried under sterile conditions (37°C) for 5–6 h. The medium in BV2 cultures was replaced with fresh one 1 h before seeding glioblastoma cells onto the inserts, then LN18 and U87 glioblastoma cells were seeded at 2×10^4^/insert on Matrigel-covered membranes in a serum-reduced medium (2% FBS). Untreated glioblastoma cells co-cultured with or without BV2 cells served as positive and negative controls, respectively. Cells were treated with the arginase-1 inhibitors OAT-90, OAT-1617 and OAT-1746 solved in PBS. The cultures were kept in incubator at 37°C with humidified air containing 5% CO_2_. After 18 h cells were fixed in ice-cold methanol and cell nuclei stained with DAPI (4′,6-Diamidino-2-Phenylindole; 1 µg/mL, Sigma). The membranes from Transwell^®^ inserts were cut out and images were acquired using a fluorescence microscope (Leica DM4000B, 10x lens) from the 5 independent fields (bottom; top; left; right side and the middle). Numbers of invading cells were counted using the ImageJ software (NIH, Bethesda, MD, USA). All experiments were performed three times, in duplicates.

### Viability Assay

Cell viability was assessed using MTT metabolism assay (U87 and BV2 cells) or MTS CellTiter 96^®^ AQueous One Solution Cell Proliferation Assay (Promega) for the primary murine microglia cultures. Cells were cultured either in 96-well plates (U87 at density of 1×10^4^) or 24-well plates (BV2 at density of 4×10^4^ and primary microglia 8×10^4^) with the indicated concentrations of the inhibitor or H_2_O (vehicle) for 24 h. MTT solution (Sigma Aldrich) was added to each well to a final concentration of 0.5 mg/mL. After 1 h of incubation at 37°C, water-insoluble dark blue formazan crystals were dissolved in DMSO. Optical densities (OD) were measured at 570 and 620 nm using a scanning multiwell spectrophotometer. The MTT assay was performed according to the manufacturer’s protocol. All measurements were carried out on three independent cell passages, in triplicates.

### Animals

Male C57BL/6J mice (10-12 weeks at the beginning of the study) were housed with free access to food and water, on a 12h/12h day and night cycle. All efforts have been made to minimize the number of animals and animals suffering. All research protocols conformed to the Guidelines for the Care and Use of Laboratory Animals (European and national regulations 2010/63/UE September 22, 2010 and Dz. Urz. UE L276/20.10.2010, respectively). Animals were decapitated by a qualified researcher. The First Warsaw Local Ethics Committee for Animal Experimentation approved the study (approval no. 562/2018).

### Determining Plasma L-Arginine and Drug Concentration

Blood plasma, as well as the brain samples (from control and treated animals), were prepared for liquid chromatography coupled with mass spectrometry (LC-MS) by homogenization in 5% trichloroacetic acid (TCA) and the concentration of L-arginine – the substrate of arginase – was determined. In the urea cycle, arginase cleaves arginine to produce urea and ornithine. Ornithine reacts with carbamoyl phosphate to form citrulline. The brain homogenates prepared for arginine measurements, as well as plasma samples were also analyzed by LC-MS to determine the concentration of the drug, which was administered to animals 2 h before the euthanasia.

### Stereotactic Implantation of Glioma Cells

Mice were deeply anesthetized with isoflurane. After identifying the sagittal and coronal sutures on the right side, a hole was drilled at the following coordinates: 1 mm anterior and 1,5 mm lateral from bregma. GL261 tdTomato^+^luc^+^ glioma cells (80,000 in 1 μL of DMEM) were stereotactically injected with a Hamilton syringe to the right striatum of the mouse 3 mm deep from the surface of the brain. The skin incision was closed and mice were monitored until they completely recovered from anesthesia. Mice were randomly allocated to the study groups. The animals were weighed weekly and observed daily for clinical symptoms and evidence of toxicity by evaluating their eating, mobility, weight loss, hair loss, and hunched posture. OAT-1746 was administered by oral gavage at 50 mg/kg twice a day from day 1 after implantation. Anti-PD-1 antibody (Biolegend, GoInVivo™ Purified anti-mouse CD279) was injected intraperitoneally (i.p.) at a dose of 2.5 mg/kg on days 8, 10, 12, 14 post-implantation. Control groups received vehicle (saline) twice a day by gavage. Animals were euthanized when they lost more than 20% of body weight compared to day 0.

### Bioluminescence Imaging

To monitor tumor growth, mice were injected i.p. with 150 mg/kg body weight luciferin (D-luciferin sodium salt BC218 Synchem) and left for 8 min. Then, the animals were anesthetized with 3% isoflurane and transferred to the X-treme Imaging System (Bruker, Germany). At 10 min after the D-luciferin injection, a photonic emission was imaged. Tumors were visualized at days 14, 21 and 28 after implantation and bioluminescent images were quantified as photon/sec/mm^2^. We applied the same ROI rectangle to all images (the whole head). Then we exported sum values for all images.

### Cytokine Analysis

Measurement of pro- and anti-inflammatory cytokines was performed in blood from control and treated animals. Blood was quickly collected to EDTA containing tubes before perfusion and centrifuged (10,000 × g) for 10 min at room temperature. The plasma was collected and stored at -80°C. The levels of cytokines were measured using the Milliplex Kit (Merk-Millipore, Germany) according to the protocol. Cytokine levels were determined using the MAGPIX Multiplexing Instrument (Luminex, TX, USA) with XPonent software and analyzed with Milliplex Analyst 5.1 software. Results were expressed as pg/mL for each cytokine.

### Immunohistochemistry on Brain Slices

The animals were sacrificed on the day 21 after GL261 tdTomato^+^luc^+^ cell implantation and perfused with 4% paraformaldehyde in PBS. Brains were removed, post-fixed for 48 h in the same fixative solution and placed in 30% sucrose in PBS at 4°C until the tissue sunk to the bottom of the flask. Tissue was frozen in Tissue Freezing Medium (Jung; Nussloch, Germany) and cut in 12 µm coronal sections using a cryostat. The slides were dried at room temperature for 2 h after being transferred from the -80°C storage. Cryosections were blocked in PBS containing 10% donkey serum and 0.1% Triton X-100 for 2 h and incubated overnight at 4°C with rabbit anti-Iba-1 and goat anti-Arg1 or with rabbit anti-CD8 antibodies. Next, sections were washed in PBS and incubated with corresponding secondary antibodies for 2 h at room temperature. All antibodies were diluted in 0.1% Triton X-100/PBS solution containing 3% donkey serum. Nuclei were counterstained with DAPI (1 µg/mL). Images were obtained using the Olympus microscope (Fluoview, FV10i). For reagent specifications, catalogue numbers, and concentrations, see the **Supplementary Table 1**. To quantify the tumor size, sections were stained with toluidine blue, and images were acquired using a Leica DM4000B microscope (Leica Microsystems, Wetzlar, Germany). Tumor areas were measured using ImageJ software (NIH, Bethesda, MD, USA) on every sixth brain slice, and tumor volumes were calculated as previously described (26).

### Tissue Dissociation, Flow Cytometry and FACS Sorting

On day 28 after GL261 tdTomato^+^luc^+^ cell implantation mice were perfused transcardially with cold phosphate-buffered saline (PBS) to clear away blood cells from the brain. The tumor-bearing hemispheres were dissociated enzymatically with a Neural Tissue Dissociation Kit with papain (Miltenyi Biotec) and gentleMACS Octo Dissociator (Miltenyi Biotec), according to the manufacturer’s protocol to obtain a single-cell suspension. Next, the enzymatic reaction was stopped by the addition of Hank’s Balanced Salt Solution with calcium and magnesium (Gibco, Germany). The resulting cell suspension was filtered through 70 μm and 40 μm strainers, and centrifuged at 300 × g, 4°C for 10 min. Next, myelin was removed by centrifugation on a 22% Percoll gradient. Briefly, cells were suspended in 25 mL Percoll solution (18.9 mL gradient buffer containing 5.65 mM NaH_2_PO_4_H_2_O, 20 mM Na_2_HPO_4_2(H_2_O), 135 mM NaCl, 5 mM KCl, 10 mM glucose, 7.4 pH; 5.5 mL Percoll (GE Healthcare, Germany); 0.6 mL 1.5 M NaCl), overlayered with 5 mL DPBS (Gibco) and centrifuged for 20 min at 950 × g at 4°C, without acceleration and brakes. Next, cells were collected, washed with PBS and counted using NucleoCounter (Chemometec, Denmark).

Samples were handled on ice or at 4**°**C without light exposure. Prior to staining with antibodies, samples were incubated with LiveDead Fixable Violet Dead Cell Stain (ThermoFisher) in PBS for 10 min to exclude nonviable cells. Next, samples were incubated for 10 min with rat anti-mouse CD16/CD32 Fc Block™ (BD Pharmingen) in Stain Buffer (BD Pharmingen) to block FcγRIII/II and reduce unspecific antibody binding. Then, cell suspensions were incubated for 30 min with an antibody cocktail in Stain Buffer (BD Pharmingen). For flow cytometry analysis of cell surface antigens the following anti-mouse antibodies were used: CD45 (30-F11), CD11b (M1/70) from BD Pharmingen and CD3 (REA641), NK 1.1 (PK136) from Miltenyi Biotec. For FACS sorting the cells were stained with CD11b (M1/70) antibody labeled with FITC (BD Pharmingen).

All antibodies were titrated prior to staining to establish the amount yielding the best stain index. Data were acquired using a BD LSR Fortessa Analyzer cytometer and analyzed with FlowJo software (v. 10.5.3, FlowJo LLC, BD). Gates were set based on FMO (fluorescence minus one) controls and back-gating analysis. Percentages on cytograms were given as the percentage of a parental gate. CD11b^+^ cells were FACS sorted using Cell Sorter BD FACSAriaII. All flow cytometry experiments were performed at the Laboratory of Cytometry, Nencki Institute of Experimental Biology. For reagent specifications, catalog numbers and dilutions see the **Supplementary Table 1**.

### RNA Isolation, mRNA Library Preparation and RNA-Sequencing

Immediately after sorting, CD11b^+^ cells were centrifuged and lysed for further isolation of RNA using the RNeasy Plus Mini Kit (Qiagen, Germany). The integrity and quality of RNA were assessed on an Agilent 2100 Bioanalyzer with an RNA 6000 Pico Kit (Agilent Technologies, CA, USA). A total of 9 strand-specific RNA libraries were prepared for sequencing (2-3 biological replicates/treatment) using a KAPA Stranded mRNA-Seq Kit (Kapa Biosystems, MA, USA). Poly-A mRNAs were purified from 100 ng of total RNA using poly-T-oligo-magnetic beads (Kapa Biosystems, MA, USA). mRNAs were fragmented and a first-strand cDNA was synthesized using reverse transcriptase and random hexamers. A second-strand cDNA synthesis was performed by removing RNA templates and synthesizing replacement strands, incorporating dUTP in place of dTTP to generate double-stranded (ds) cDNA. dsDNA was then subjected to addition of “A” bases to the 3′ ends and ligation of adapters from NEB, followed by uracil digestion by USER enzyme (NEB, MA, USA). Amplification of fragments with adapters ligated on both ends was performed by PCR using primers containing TruSeq barcodes (NEB, Ipswich, MA, USA). Final libraries were analyzed using Bioanalyzer and Agilent DNA High Sensitivity chips (Agilent Technologies, Santa Clara, CA, USA) to confirm fragment sizes (~300 bp). Quantification was performed using a Quantus fluorometer and the QuantiFluor dsDNA System (Promega, Madison, Wisconsin, US). Libraries were loaded onto a rapid run flow cell at a concentration of 8,5 pM onto a rapid run flow cell and sequenced on an Illumina HiSeq 1500 paired-end.

### Data Processing and Analysis

Illumina-specific adapters, short reads, and low quality 5’ and 3’ bases were filtered out in the FASTQ format files using Trimmomatic (27) tool (version 0.36). The resulting RNA sequencing reads were aligned to a reference mouse genome sequence (mm10) with STAR aligner (28) (version 2.6.1b) using the two pass Mode Basic option. Duplicate reads were then identified and flagged using Picard Tools (version 2.17.1) [broadinstitute.github.io/picard/]. Quantification of mapped reads and summarization by gene was performed using HTSeq-count (29) (version 0.11.1), with paired mode (-p) and reverse stranded mode (-s reverse) enabled, and only reads with MapQ values of 10 or higher were considered. Low-expressed features were filtered out and an analysis of differentially expressed genes was performed using DESeq2 (version 1.24.0) (30). Only mRNAs encoding protein-coding genes were retained for downstream analysis.

To identify transcriptomic differences between groups, differential expression analysis was performed using DESeq methods, with the control state (CTR) as the reference group and compared with the OAT-1746, anti-PD-1 and combination groups. The variance stabilizing transformation (vst function) was used for visualization. Pathway enrichment analysis was performed by selecting statistically significant genes (adjusted p-values ≤ 0.05) and correcting type I errors in multiple testing using the Bonferroni-Hochberg (BH) method. Gene Ontology Biological Processes (GO: BP) was used to better understand the mechanistic findings of the enriched gene lists. The clusterProfiler (31) and VennDiagram (32) packages were used to visualize the results.

## Results

### *ARG1* Expression Is Highly Upregulated in Human Glioblastoma Samples and in Murine Experimental Gliomas

**Figure 1:**
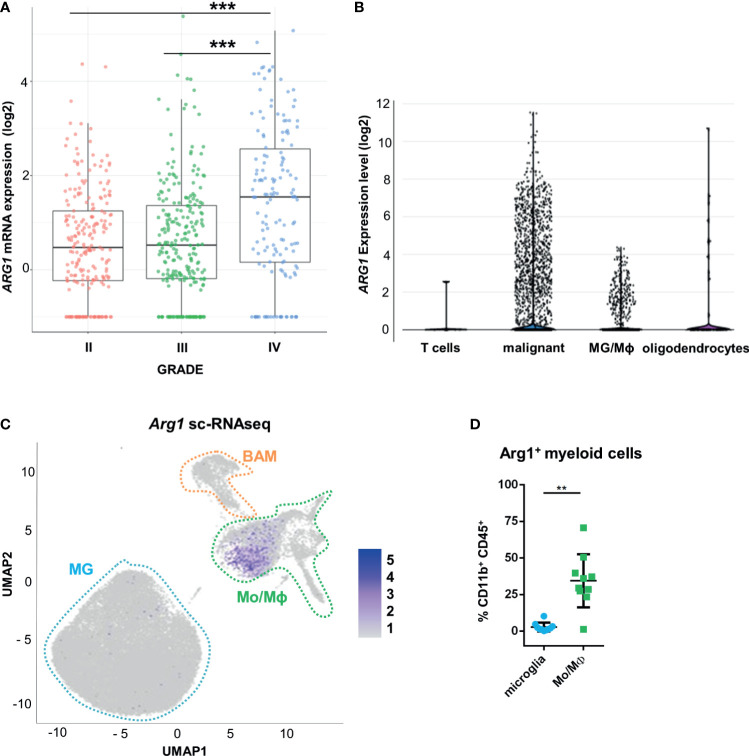
*ARG1* expression is highly upregulated in human glioblastomas and murine experimental gliomas. **(A)** *ARG1* expression in gliomas of different WHO grades (WHO grades II- IV) in TCGA datasets. Statistical significance was determined by Tukey’s Honest Significant Difference (HSD). ***p < 0.001. **(B)** Expression of *ARG1* in malignant cells and microglia/macrophages (MG/MΦ) in 10 samples of astrocytomas in single-cell RNA-seq datasets (public data) from (33). **(C)** UMAP plot of CD11b^+^ cells from GL261 gliomas (n=8). Projection of cells combined from clusters identified as microglia, monocytes/macrophages (Mo/MΦ), and BAMs. Plots depicting *Arg1* mRNA which is highly expressed in infiltrating Mo/MΦ. **(D)** Flow cytometry analysis of Arg1 expressing cells among microglia (CD11b^+^CD45^lo^) and Mo/MΦ (CD11b^+^CD45^high^) cells sorted from murine gliomas (n=10); Wilcoxon matched-pairs signed rank, two-tailed, **p < 0.01.

Using transcriptomic data from The Cancer Genome Atlas (TCGA), we examined *ARG1* and *ARG2* expression in human gliomas of different WHO grades II, III, IV. The highest mRNA levels of both genes were found in GBM samples (**Figure 1A** and **Supplementary Figure 1A**). To determine a cell source of *ARG1* and *ARG2* expression, we explored the single-cell RNA sequencing (scRNA-seq) data from 10 astrocytoma samples (33) and checked the gene expression in various cell populations from the tumors using the SingleCell data portal (https://singlecell.broadinstitute.org/). High expression of *ARG1* and *ARG2* was detected in malignant cells and tumor-infiltrating microglia/macrophages (MG/MΦ) (**Figure 1B** and **Supplementary Figure 1B**). We took advantage of having in-house sc-RNA-seq data of CD11b^+^ immunosorted from murine GL261 gliomas, which provided resolution to distinguish resident microglia from CNS-border associated macrophages (BAMs) or monocytes/macrophages (Mo/MΦ) (34). Using these data, we analyzed *Arg1* and *Arg2* expression in the discrete myeloid subpopulations. There was a low number of microglial cells expressing either *Arg1* (< 1%) or *Arg2* (< 0.1%) mRNA and both genes were more abundantly expressed in the Mo/MΦ population (11% and 6%, respectively) (**Figure 1C** and **Supplementary Figure 1C**). *Arg1* expression levels were significantly higher than *Arg2* mRNA levels both in microglia and Mo/MΦ immunosorted from tumor-bearing brain (**Supplementary Figure 1D**). Additionally, we assessed Arg1 levels by flow cytometry in CD11b^+^ cells isolated from tumor-bearing hemispheres at day 21 post-implantation (**Figure 1D**). The percentage of Arg1^+^ cells was higher in Mo/MΦ infiltrating from the periphery (CD11b^+^CD45^high^) than in resident microglia (CD11b^+^CD45^low^), which corroborated the results from scRNA-seq analysis (**Supplementary Figure 1D**). Overall, these results confirm high *ARG1* and *ARG2* expression in human malignant cells and glioma-infiltrating monocytes/macrophages. Arg1 is a predominant isoform expressed in myeloid cells in the brain of tumor-bearing mice.

### The Effect of OAT Inhibitors on Human Arginase 1/2 Activity and Glioma Cell Invasion

We have previously demonstrated that *Arg1* mRNA is upregulated in microglia exposed to glioma during reprogramming of microglia into tumor-supportive, immunosuppressive cells (25, 35). Arginase inhibitors OAT-1746 and OAT-1617 were designed and synthesized by OncoArendi Therapeutics, Warsaw. OAT-1746 inhibited ARG1/2 at low nanomolar concentrations, reversed ARG1-inhibited proliferation of human and murine T cells and showed significant antitumor efficacy in various non-CNS tumor models (36).

**Figure 2:**
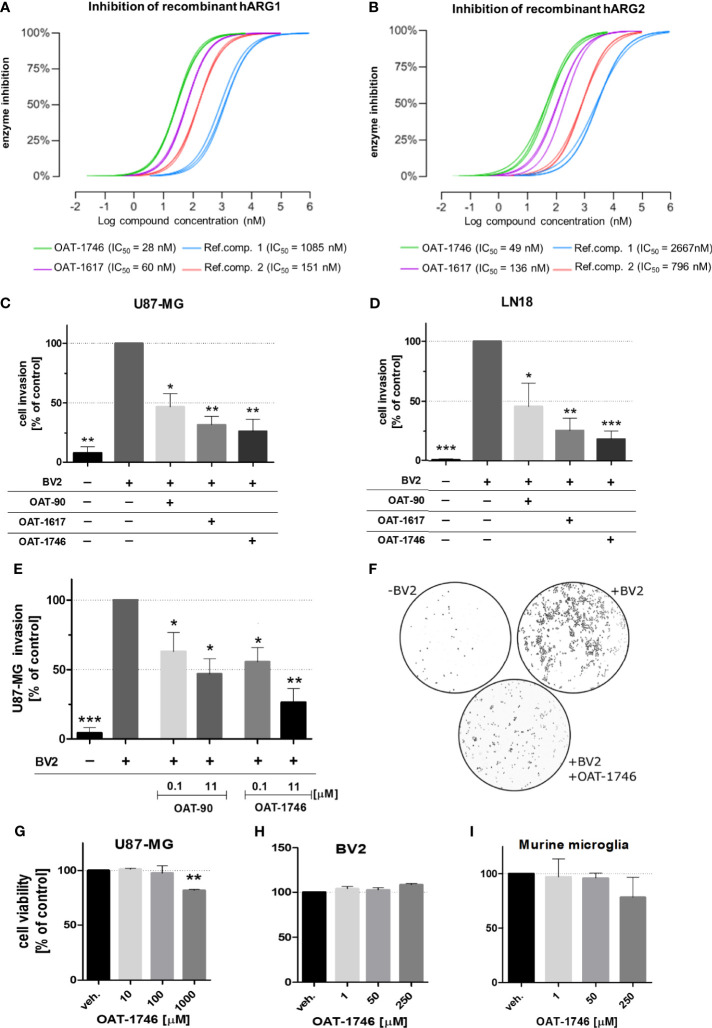
The effect of OAT inhibitors on human arginase 1/2 activity and microglia-induced glioma invasion. **(A, B)**. The activity of two new and two reference ARG inhibitors was tested towards recombinant human ARG1 and ARG2 (hARG1 and hARG2, respectively) The IC values were determined. **(C, D)** Graphs represent relative invasion of **(C)** U87-MG or **(D)** LN18 cells induced by the co-culture with murine microglial cells (BV2). Tumor invasion was determined using a Matrigel matrix assay. Invasion of glioma cells co-cultured with BV2 cells is set as 100%. Three tested inhibitors (OAT-90, OAT-1617, OAT-1746) were used at 11 µM concentration and all of them effectively reduced glioma invasion. Data are expressed relatively to a basal invasion in the absence of microglial cells. **(E)** The effects of OAT-90 and the second generation inhibitor OAT-1746 applied at 0.1 and 11 µM concentration on microglia induced invasion of U87-MG cells. Data are presented as means ± S.D and were calculated from three independent biological experiments. Statistical significance was evaluated using one-sample t-test. **(F)** The representative images of DAPI-stained U87-MG glioma cells on inserts show the nuclei of invading cells in the presence or absence of BV2, and OAT-1746. **(G, I)** The effect of OAT-1746 on cell viability was determined using MTT metabolism test. Cells were incubated for 24 h with or without OAT-1746 at given concentrations. The influence of OAT-1746 on the viability of **(G)** U87-MG human glioma cells, **(H)** BV2 microglial cells and **(I)** primary murine microglia was determined. Data are presented as means ± S.D (n=3 independent biological experiments). Significance of differences between the treatments was evaluated using one-way ANOVA followed by Dunnett’s post-hoc test, p-Values were considered as significant when ***p < 0.001; **p < 0.01; *p < 0.05.

OAT-1746 and OAT-1617 as well as two reference compounds were tested in biochemical assays for the ability to inhibit arginase activity and in cellular assays for the ability to block tumor cell invasion. OAT-1746 inhibited recombinant human ARG1 activity (IC_50_=28 nM) and the related enzyme ARG2 (IC_50_=49 nM) better than two reference compounds (**Figures 2A, B** **)**. ARG2 catalyzes an identical chemical reaction and exhibits 60% sequence identity with ARG1 (37).

To evaluate the effects of these novel arginase inhibitors on glioma invasion, we performed a Matrigel invasion assay using two human glioma cell lines: U87-MG **(** **Figure 2C**) and LN18 (**Figure 2D**). Immortalized BV2 microglial cells, similarly to primary microglial cultures, support glioma invasion (38). In co-cultures, glioma invasion was strongly induced in the presence of BV2 microglial cells and all three arginase inhibitors significantly decreased the proportion of invading cells. OAT-1746 at a concentration of 11 µM reduced microglia-induced invasion of glioma cells more efficiently than the reference compound OAT-90 and the older generation inhibitor OAT-1617. The inhibitory effect of OAT-1746 on glioma invasion was concentration-dependent, whereas this dependence was not observed for OAT-90 (**Figure 2E**). Representative images from Matrigel invasion assays show an increased number of invading glioma cells in co-cultures with BV2 cells and the inhibitory effect of 11 µM OAT-1746 (**Figure 2F**). These results demonstrate that the inhibition of arginase activity reduced microglia-dependent invasion of human glioma cells.

To assess the potential toxicity of OAT-1746, we determined the effects of increasing drug concentrations on cell viability by performing MTT metabolism assays on human glioma cells (**Figure 2G**), murine microglial BV2 cells (**Figure 2H**), and murine primary microglia cultures (**Figure 2I**). The OAT-1746 inhibitor was not toxic towards the tested cells at concentrations up to 1000 µM; a decrease in the viability of U87-MG cells was observed at the highest concentration, which exceeds by over two folds of magnitude the effective IC_50_ concentration of the inhibitor. The results provide evidence for the efficacy of the new compound and its safety at therapeutically relevant concentrations.

### OAT-1746 Treatment Increases Arginine Levels in the Brain and Plasma but Does Not Show an Antitumor Activity

To study the antitumor activity of the arginase inhibitor OAT-1746, we employed a syngeneic model of GL261 mouse glioma cells implanted into immunocompetent C57BL/6J mice. GL261 glioma cells were stably transfected with constructs allowing the expression of a red fluorophore tdTomato and luciferase to visualize tumor growth using *in vivo* imaging. Tumor-bearing mice received OAT-1746 (50 mg/kg) or saline twice a day by oral gavage.

**Figure 3:**
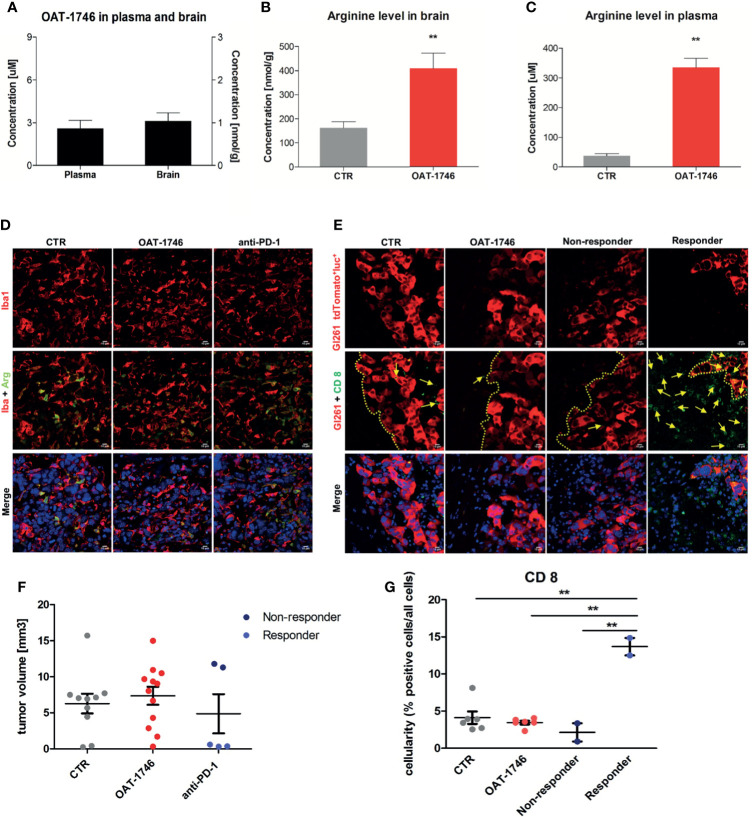
Treatment with OAT-1746 increases arginine levels in the brain and plasma but has no antitumor activity against murine GL261 gliomas. **(A–C)** Tumor-bearing mice were treated with OAT-1746 twice a day and samples were collected 2 h after the last dosing (n = 6 per group). Concentrations of OAT-1746 **(A)** and L-arginine in tumor-bearing brains **(B)** and plasma **(C)** from the same animals were measured by LC/MS at day 14 post-implantation. The results were compared using the non-parametric Mann-Whitney test. **p < 0.01. **(D)** Mice with implanted GL261 tdTomato^+^luc^+^ glioma cells received orally saline CTR (n=12) or OAT-1746 50 mg/kg twice a day for 21 days (n=12). A separate group received anti-PD-1 antibody (2.5 mg/kg, i.p.) at days 8, 10, 12 and 14 (n=5). Representative images of the glioma-bearing brains (at day 21) stained with anti-Iba1 (in red) and anti-Arg1 (in green) antibodies, and co-stained with DAPI are shown. **(E)** Representative confocal microscopy images of CD8^+^ T and NK cells within the tumors after OAT-1746 or anti-PD-1 antibody administration. The yellow line separates tumor areas (with glioma cells showing red fluorescence) and the parenchyma border; cell nuclei are counterstained with DAPI (blue); magnification x60. Yellow arrows indicate the accumulation of CD8^+^ cells (in green) in the responder PD-1 inhibition but not in the non-responder. **(F)** Quantification of tumor volumes at day 21 post-implantation. Each individual from CTR (n=12), OAT-1746 (n=12) and anti-PD-1 (n=5) groups is shown. In the checkpoint inhibitor treatment group non-responders (n=2) and responders (n=3) are marked in dark and light blue, respectively. Tumor areas were measured using ImageJ in every sixth brain slice, and tumor volumes were calculated; the mean ± SEM, p values were calculated using the Mann–Whitney U-test. **(G)** Quantification of CD8^+^ cells related to the total number of cells in the area of interest. The cells were counted using ImageJ software and average values from 5 fields are presented. Significance was calculated with One-Way ANOVA, Bonferroni’s multiple comparison test was used to compute p values (CTR n=6; OAT-1746 n=6, anti-PD-1 n=4); **p < 0.01.

First, we determined if the arginase inhibitor crossed the blood-brain-barrier (BBB) by measuring directly the level of the drug as well as the level of L-arginine - a substrate of arginase, in the brains and sera of mice after 14 days of OAT-1746 treatment. The brain and plasma were taken 2 h after the last drug administration. OAT-1746 was detected in the brain extracts, which confirms that it crosses the BBB and accumulates in the brain (**Figure 3A**). The administration of OAT-1746 resulted in an increase of arginine concentration, both in the brain (**Figure 3B**) and the blood plasma (**Figure 3C**).

To study the effects of arginase inhibition on the immune cells in the TME and visualize the infiltration of these cells into tumors, we performed immunohistochemical double staining for Arg1 and Iba1 (a marker of microglia/macrophages), and for CD8 (a marker of cytotoxic T cells, present also on NK cells). Mice were treated with OAT-1746 at 50 mg/kg twice a day. In parallel, we evaluated the effect of anti-PD-1 antibody which was injected intraperitoneally on days 8, 10, 12 and 14 post-implantation. The administration of OAT-1746 or anti-PD-1 treatment did not change the accumulation of Iba1^+^ and Arg1^+^ cells in experimental gliomas (**Figure 3D**). CD8^+^ cells were distinctly located at the invasive tumor margin and more CD8^+^ cells were detected in animals with smaller tumors (**Figure 3E**). As tumor cells displayed red fluorescence, evaluation of brain sections allowed quantification of a tumor growth. OAT-1746 treatment did not reduce the tumor growth when compared to the control group. However, among anti-PD-1 treated animals we noticed two groups with different tumor sizes, which is consistent with a division into responders and non-responders observed in patients (**Figure 3F**). Quantification of CD8^+^ T cell densities at the invasive tumor margin showed an increased number of CD8^+^ cells in responders when compared to non-responders (**Figure 3G**). Non-responders had also a lower density of CD8^+^ T cell than OAT-1746-treated animals. These findings show that the arginase inhibitor alone is not capable of inhibiting glioma growth and anti-PD-1 treatment induces the response in a half of animals.

### Combined OAT-1746 and Anti-PD-1 Treatment Reduces Glioma Growth

**Figure 4:**
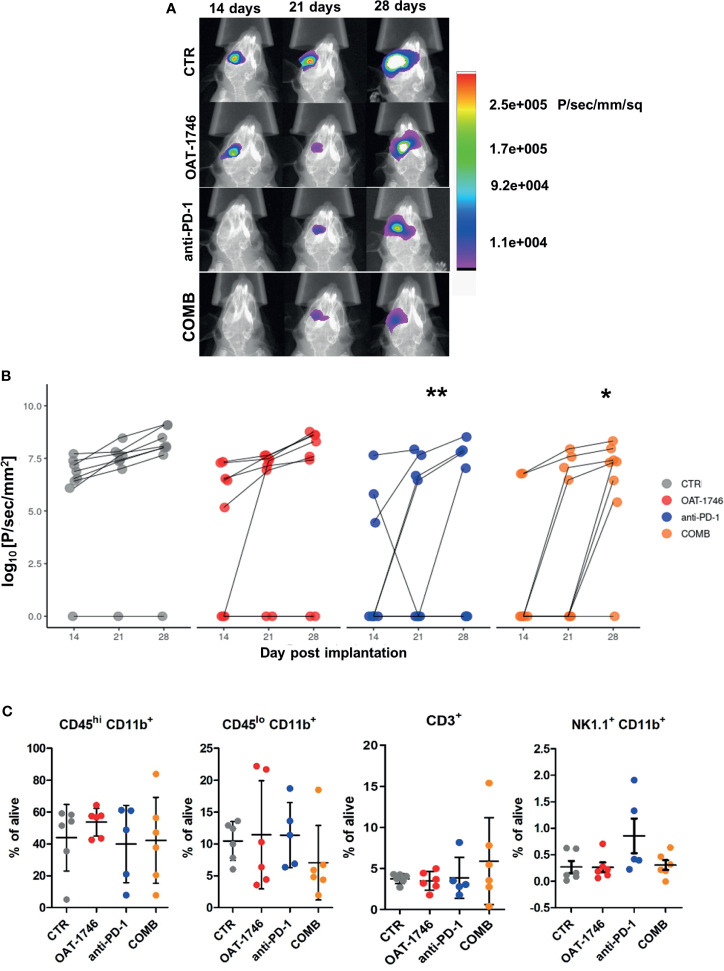
Combined OAT-1746 and anti-PD-1 treatment reduces glioma growth. Mice were implanted with GL261 tdTomato^+^luc^+^ glioma cells and received saline (CTR), OAT-1746 (twice a day) alone or anti-PD-1 antibody at day 8, 10, 12 and 14 alone or in combination (COMB). **(A)** Representative images of tumor bioluminescence with Bruker Xtreme imaging. Color intensity represents a relative luciferase signal. Bioluminescence signals are plotted as photon/sec/mm^2^ against time at indicated days post-implantation. **(B)** Tumor size measured using *in vivo* bioluminescence imaging at various times post-implantation. The effect of treatment and time on tumor progression was assessed with factorial ANOVA; treatment effect F_3,89_= 4.801, p=0.004, day post-implantation effect F_2,89_ = 4.726, p=0.011, and Tukey HSD *post hoc* test: CTRL-OAT p_adj_=0.455, CTRL-CHECK p_adj_=0.005, CTRL-COMB p_adj_=0.021, COMB-OAT-1746 p_adj_=0.46, COMB-anti-PD-1 p_adj_=0.96. **(C)** At day 28 post-implantation animals were perfused with PBS, control and tumor-bearing brains were removed and processed to isolate myeloid cells by FACS. Percentages of peripheral macrophages (CD11b^+^CD45^hi^), microglia (CD11b^+^CD45^lo^), CD3^+^ and NK1.1^+^ cells were evaluated. Significance of differences between groups was assessed with One-Way ANOVA followed by Bonferroni’s multiple comparison test. p-Values were considered as significant when **p < 0.01; *p < 0.05.

Antitumor immunity can be blocked by more than one suppressive mechanism, including the expression of immune checkpoint proteins and the depletion of essential nutrients from TME (39). We assumed that combining OAT-1746 with an immune-modulating agent, such as anti-PD-1 antibody, might improve drug efficacy. Tumor growth was monitored by measuring luminescence signal 14, 21 and 28 days after implantation of GL261 tdTomato^+^luc^+^ glioma cells. Representative images of gliomas at different time points are shown (**Figure 4A**). While OAT-1746 alone did not show any effect on the tumor volume and the anti-PD-1 treated animals were split into responders and non-responders, the combined treatment resulted in significantly reduced glioma growth at day 28. The combination of OAT-1746 with anti-PD-1 delayed, and in some cases abrogated, tumor growth. Effects of treatment and time of tumor progression were calculated with multifactorial ANOVA (**Figure 4B**). Both the “treatment” effect F_3,89_ (4.801)=0.004 and the “time after implantation” effect F_2,89_(4.726)=0.011 were significant. Tukey’s honest significant difference (HDS) *post hoc* test was used to compare experimental groups and p values were as follows: CTR-OAT-1746 p=0.455, CTR-anti-PD-1 p=0.005, CTRL-COMB p=0.021, COMB-OAT-1746 p=0.46, COMB-anti-PD-1 p=0.96. These results provide evidence that combining arginase inhibition with targeting immune checkpoints could be an effective strategy to reduce glioma growth. Administration of the drug delayed tumor growth. Inhibition of glioma growth was augmented when two agents were combined. At day 14 only 2 out of 8 animals developed tumors in the COMB group, while there were 5 out of 8 mice with tumors in OAT-1746-treated cohort.

To further investigate the immune cell-mediated mechanism of action of OAT-1746 and anti-PD-1, flow cytometry was performed on cells isolated from tumors, and changes in specific immune cell populations were quantified. Gating strategy is shown in the **Supplementary Figure 2**. The administration of OAT-1746 or anti-PD-1 treatment did not change the percentage of microglia (CD11b^+^CD45^low^) and blood-derived macrophages (CD11b^+^CD45^high^) in experimental gliomas. We noticed an increase in NK cells, however it did not reach statistical significance in anti-PD-1-treated animals compared to control group. An increased percentage of CD3^+^ T cells was observed in the majority of animals in the COMB group (**Figure 4C**). These results provide evidence that combining arginase inhibition with targeting the immune checkpoint, could be an effective strategy to improve immunotherapy outcome.

**Figure 5:**
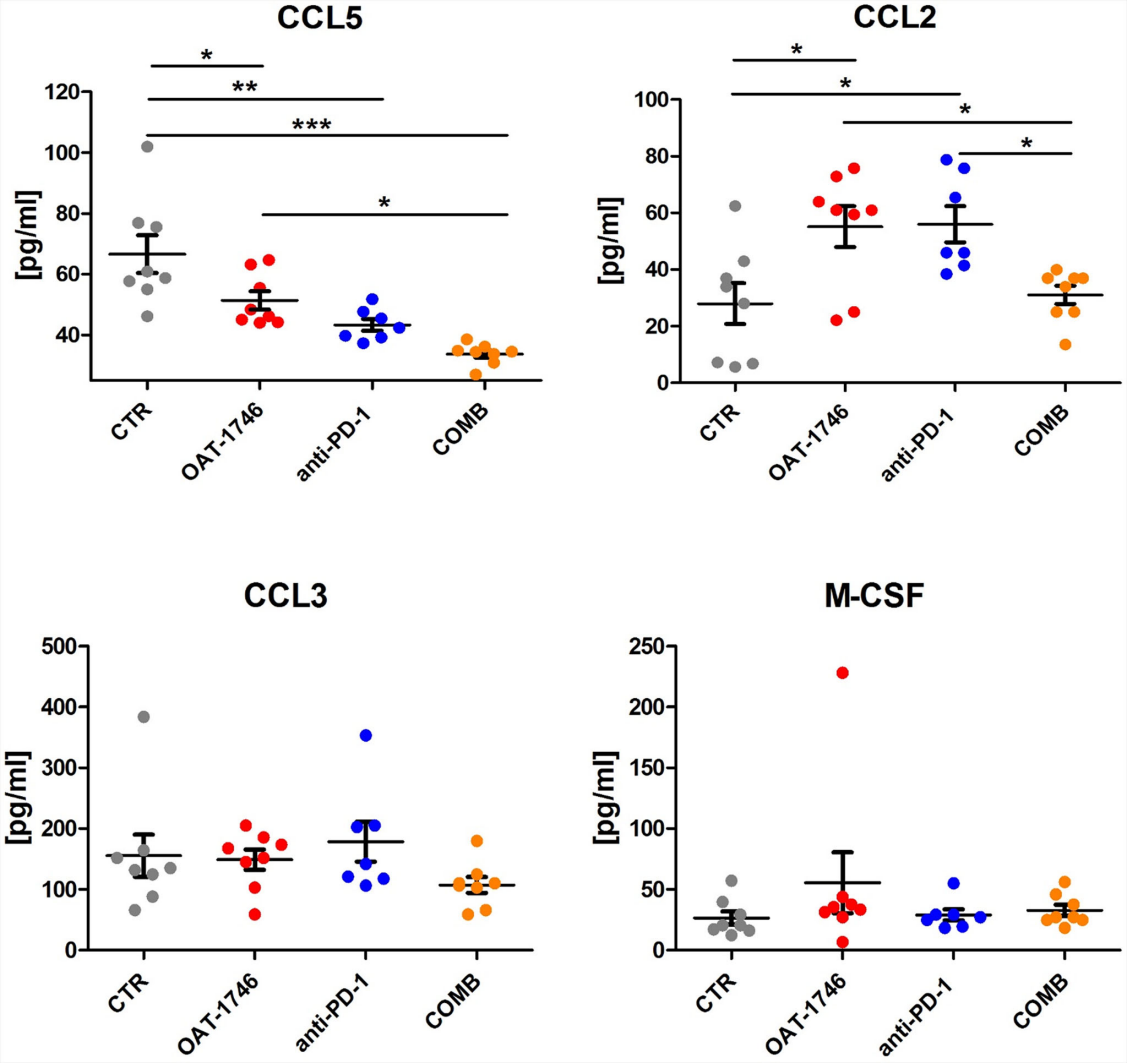
The effect of treatment on the levels of pro- and anti-inflammatory cytokines. The levels of pro/anti-inflammatory cytokines were determined in blood plasma of the animals from the experimental groups described above using a multiplexed bead-based assay and Luminex technology (MAGPIX). Histograms show the levels of tested cytokines in pg/mL; the results are shown as means ± SEM; significance was assessed with One–Way ANOVA and Bonferroni’s multiple comparison test, ***p < 0.001; **p < 0.01; *p < 0.05.

To obtain more insights regarding the enhancement of immunotherapy, we determined the levels of pro- and anti-inflammatory cytokines in the blood (plasma) collected 2 h after the last administration of OAT-1746. The levels of CCL5 (C-C Motif Chemokine Ligand 5) were reduced in both OAT-1746 or anti-PD-1 treated animals but the observed decrease was much stronger in animals treated with the combination of two agents (**Figure 5**). Interestingly, both OAT-1746 and anti-PD-1 treated animals showed higher levels of the cytokine CCL2 and this effect was abrogated in animals receiving the combination (**Figure 5**). The levels of CCL3 and M-CSF (macrophage colony stimulating factor, Csf1) were not changed by the treatments. C–C motif chemokine ligand 2 (CCL2) and CCL5 are the main chemokines involved in monocyte migration to tumors (40). CCL2 *via* its receptor CCR2 controls the migration of regulatory T cells (Treg) and myeloid suppressor cells (41), as well as their ability to promote tumor growth (42). The CCL5/CCR5 axis directs infiltration and interactions between monocytes/macrophages and mesenchymal stem cells. CCR5 is highly expressed in glioblastoma, controls glioma invasion and its expression is associated with the poor prognosis of GBM patients (43, 44).

### Transcriptomic Profiles of CD11b^+^ From OAT-1746 Treated Animals Show Reduced Expression of Tumor Supportive Genes

While OAT-1746 treatment alone showed no antitumor activity, it increased the therapeutic efficacy of PD-1 inhibition in murine gliomas. To gain insights into potential mechanisms of the observed phenomenon, we compared the transcriptomic profiles of glioma-associated CD11b^+^ cells isolated from control mice (CTR), mice treated with anti-PD-1, OAT-1746 or a combination of both (COMB). CD11b^+^ cells were sorted from tumor-bearing hemispheres as previously described (34) and the gating strategy is shown in the **Supplementary Figure 3**. CD11b^+^ cells immunosorted from GL261 tumor-bearing hemispheres encompass microglia, infiltrating monocytes/macrophages and BAMs, as well as granulocytes, certain subpopulations of dendritic cells and NK cells (34). RNA sequencing of total RNA isolated from CD11b^+^ cells was followed by computational analyses of differentially expressed genes and detection of altered signaling pathways.

**Figure 6:**
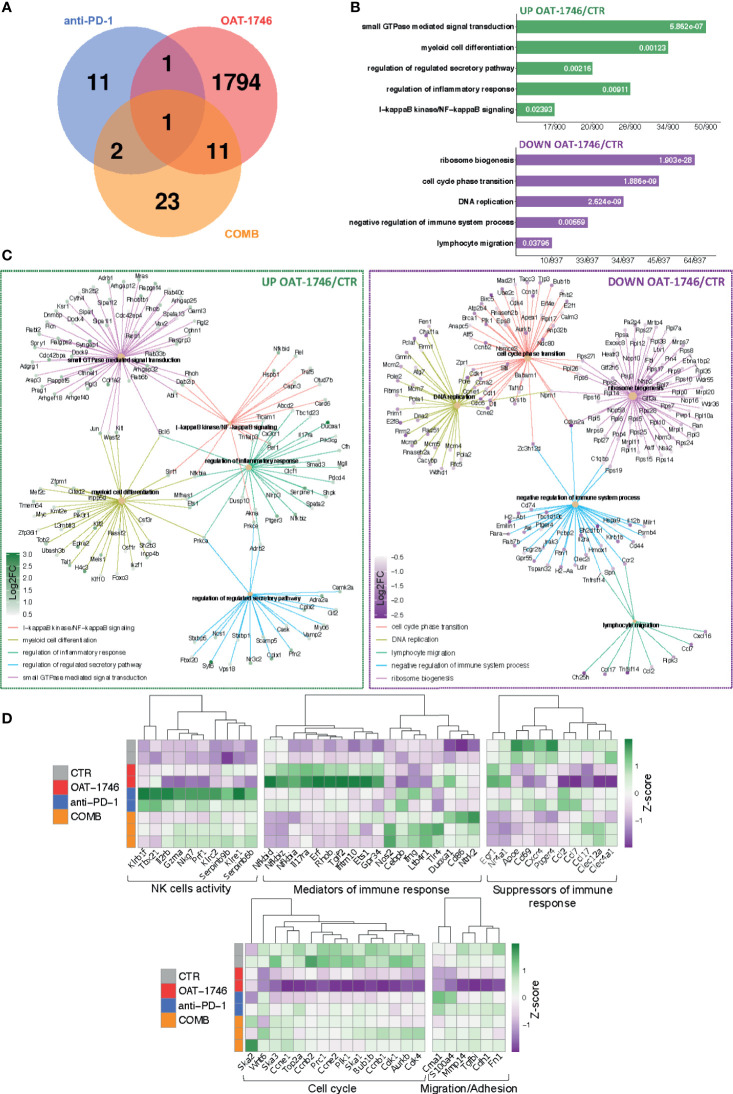
Transcriptomic profiles of CD11b^+^ from OAT-1746-treated mice show reduction of the pro-tumor phenotype genes and upregulation of gene expression indicative of antitumor responses. **(A–D)** Gene expression profiling was performed by RNA-seq of CD11b^+^ cells from the tumor-bearing hemispheres of mice from the experimental groups at day 21 post-implantation. **(A)** Venn diagram showing the number of differentially expressed genes (DEG) between OAT-1746, anti-PD-1 and COMB compared to the CTR group (p_adj_<0.05). **(B)** Functional enrichment analysis with Gene Ontology (GO) biological processes for up- and downregulated genes in OAT-1746 compared to CTR. Enriched GO pathway names are shown, the size of the bars indicates the ratio of genes (a number of genes annotated to the pathway/total number of DEGs with adjusted p-values < 0.05). **(C)** Graphical representation of selected overrepresented categories among DEGs in the OAT-1746 *versus* the CTR group **(D)** Z-score heatmaps for selected genes represent the relative change in gene expression in the CD11b^+^ cells from OAT-1746, anti-PD-1 or COMB treated animals.

OAT-1746 had the greatest impact on the transcriptome of CD11b^+^ cells and over 1800 genes were identified as differentially expressed genes (DEG) between the OAT-1746 and CTR groups (p_adj_<0.05) (**Figure 6A**). To classify the DEGs into functional categories, we performed Gene Ontology (GO) enrichment, in which genes were assigned to biological processes. Pathway enrichment for OAT-1746 showed significant terms which are shown in **Figures 6B, C**. The number of DEGs between other groups (anti-PD-1 *vs*. CTR and COMB *vs*. CTR) was low and no significantly affected processes were identified. Among the differentially up-regulated genes in the OAT-1746 group, we found a significant overrepresentation of genes involved in GTPase-mediated signal transduction, myeloid cell differentiation, NF-κB (nuclear factor kappa B) signaling, and regulation of inflammatory responses. In contrast, the downregulated genes in the OAT-1746 group were related to ribosome biogenesis, the cell cycle and DNA replication, which indicates the decreased proliferative activity of the tumor-associated myeloid cells.

We selected several DEGs which showed statistically significant changes in at least one of these different comparisons (OAT-1746 *vs* CTR, anti-PD-1 *vs* CTR or COMB *vs* CTR) and profiled their expression in CD11b^+^ cells from the tested groups (**Figure 6D**). Genes associated with differentiation and cytotoxic activity of NK cells were significantly upregulated under anti-PD-1 treatment and in combination with OAT-1746. This group included genes coding for NK cell surface proteins (*Klrb1f*, *Klre1*, *Klrc2*), cytotoxic granule protein (*Nkg7*), T-Box transcription factor 21 (*Tbx21*), which is involved in NK cell differentiation and regulation of IFNγ expression, as well as granzyme A (*Gzma*) and perforin (*Prf1*), which are the major cytolytic factors in antitumor response of cytotoxic NK and T cells. In parallel, *Serpinb6b* and *Serpinb9b*, which encode serine protease inhibitors, were upregulated, which may protect leukocytes from the cell death mediated by granzyme A and B, respectively. Such a signature indicates an increased intra-tumoral influx of activated cytotoxic NK cells.

Moreover, treatment with OAT-1746 induced the expression of genes related to NF-κB pathways (*Nfkbia*, *Nfkbid*, *Nfkbiz*) and a number of GTPases-encoding genes, including RhoB (*Rhob*), which was shown to increase NF-κB activity towards *IL-1β*, *IL-6*, and *TNF-α* genes in macrophages (45). In CD11b^+^ cells from mice treated with OAT-1746, we found high expression of *Gpr34*, which is upregulated in microglia during inflammation, *Tgif2*, which encodes a repressor of TGFβ-mediated responses, and *Duoxa1*, which encodes dual oxidase maturation factor 1 (DUOXA1) involved in pathways generating reactive oxygen species (ROS). Effective upregulation of several genes implicated in the antitumor immune response, such as those encoding for nitric oxide synthase (*Nos2*), IFNγ (*Ifng*), Tlr4 (*Tlr4*), and CD86 (*CD86*), which provides co-stimulatory signals necessary for T-cell activation, has been demonstrated in the COMB group.

Treatment with OAT-1746 resulted in a reduced expression of genes related to DNA replication and cell cycle progression, including topoisomerase 2a (*Top2a*), cyclin B1, E1 and E2 (*Ccnb1*, *Ccne1*, *Ccne2*); spindle and kinetochore-associated complex subunit 1, 2 and 3 (*Ska1*, *Ska2*, *Ska3*), as well as mitotic checkpoint kinase Bub1b (*Bub1b*) and aurora kinase B (*Aurkb*), which are involved in chromosome segregation during mitosis. This is consistent with the requirement of arginase activity for the production of L-ornithine from arginine, which is required for cell proliferation (46).

Among downregulated genes in the OAT-1746 group, we identified those involved in lymphocyte chemotaxis, i.e. *Ccl2*, *Ccl7* and *Ccl17*, and genes related to migration and invasion (*Cme1*, *S100a4* and *Mmp14*). Ccl2 and Ccl7 (also known as monocyte chemoattractant protein 1 and 3, respectively) play a critical role in the recruitment of monocytes and neutrophils to the inflamed or tumor tissue, while Ccl17 attracts regulatory T cells (47). Metalloproteinase 14 (MMP14) participates in MMP2 activation and the degradation of the extracellular matrix (ECM). MMP14 is upregulated in GAMs, and its expression correlates with the increased tumor growth in murine glioma models (48). *Clec12a* and *Clec4a1*, encoding inhibitory receptors for dendritic cells, were downregulated in the OAT-1746 group. The expression of genes encoding factors involved in the suppression of immune responses, i.e.: ApoE – a marker of an anti-inflammatory GAMs phenotype (49), CD69 – a negative regulator of immune responses implicated in inducing the exhaustion of tumor-infiltrating T cells (50), and Cxcr4 – a receptor for the immunosuppressive chemokine Cxcl12, were downregulated in the OAT-1746 and COMB groups. The decreased expression of these genes together with the upregulation of genes encoding mediators of the pro-inflammatory response suggests reprogramming of myeloid cells and restoration of the antitumor immunity.

## Discussion

Current therapies are not effective in glioblastoma patients as gross resection does not completely remove tumor cells, and the inactivation of tumor suppressors and enhanced DNA repair result in tumor cell resistance to radiotherapy and TMZ. Approximately 40% of patients with GBM do not or poorly respond to therapy and patients frequently experience fast tumor recurrence (51). While immunotherapy has been effective in many solid tumors, the results in GBM are disappointing despite the fact that PD-1 is an important checkpoint inhibitor in GBM (11). The failure of GBM to respond to anti-PD-1 is attributed to the immunosuppressive TME, which as in other “cold” tumors is characterized by a paucity of tumor infiltrating lymphocytes and a predominance of immunosuppressive myeloid cells (52–54). GAMs produce CCL2, a chemokine recruiting CCR4^+^ Treg and CCR2^+^Ly-6C^+^ monocytic MDSCs in murine gliomas (41). The combination of PD-1 blockade and CCR2 inhibition (both genetic and pharmacological with the CCR2 agonist CCX872) improved survival of KR158 glioma-bearing mice, and reduced accumulation of CD11b^+^/Ly6C^hi^/PD-L1^+^ MDSCs in gliomas. The combined treatment resulted in increased TILs infiltration, IFNγ expression and the decreased expression of exhaustion markers in CD4^+^ and CD8^+^ T cells (55).

Exploring various public datasets we found the elevated expression of *ARG1* and *ARG2* in high grade gliomas, in particular in highly aggressive GBMs. Interrogation of single-cell sequencing data from gliomas shows *ARG1* and *ARG2* expression in both malignant cells and microglia/macrophages in human high grade gliomas. Both arginase isoforms are expressed in myeloid cells in murine experimental gliomas, however *Arg1* mRNA levels are significantly higher than *Arg2*. Moreover, *Arg1*, a phenotypic marker of immunosuppressive myeloid cells, is expressed mainly in infiltrating monocytes as compared to microglia.

Newly developed ARG1/2 inhibitors showed a comparable inhibitory efficacy towards recombinant proteins *in vitro* as two reference inhibitors and several compounds described in the literature (56). In microglia-glioma co-cultures, which are used to model microglia-induced glioma invasion, OAT-1746 at micromolar concentrations strongly reduced glioma invasion, and this effect was concentration dependent. The reference compound was less effective and did not show any dose dependency. OAT-1746 up to the concentration of 1 mM was not toxic to two types of microglial cells and glioma cells, only a 20% reduction of the cell viability was detected in U87-MG glioma cells treated with this high dose of the drug. The concentration that showed toxicity exceeds by over two orders of magnitude the effective concentration of the inhibitor. The results provide evidence for the efficacy of a new compound and its safety in therapeutically relevant concentrations. Moreover, in the previous studies OAT-1746 showed no toxicity in experimental animals after multiple oral dosing in mono- or combinatorial therapies in other tumor models (36, 57). The presented data provide a strong rationale for using an ARG1 inhibitor OAT-1746 to block the pro-tumor activity of GAMs. OAT-1746 penetrated BBB and significantly increased the concentration of arginine in the brain and plasma of the receiving mice. While OAT-1746 did not affect the accumulation of microglia/macrophages (Iba1^+^ cells) and CD8^+^ cells, it considerably changed the transcriptomic profiles in CD11b^+^ immunosorted from tumor-bearing brains.

OAT-1746 had the highest impact on the transcriptome of CD11b^+^ cells in comparison to other treatments. Gene Ontology (GO) term enrichment revealed a significant overrepresentation of genes involved in GTPase-mediated signal transduction, myeloid cell differentiation, NF-κB signaling and the regulation of inflammatory responses, with simultaneous significant downregulation of genes related to ribosome biogenesis, the cell cycle and DNA replication. These changes in transcriptome are consistent with the decreased proliferation of GAMs (*Top2a*, *Ccnb1*, *Ccne1*, *Ccne2*, *Ska1*, *Ska2*, *Ska3, Bub1b, Aurkb*) and changes in their functions. High expression of inflammation mediators such as genes coding for *Gpr34* and *Duoxa1* indicates a switch to the pro-inflammatory phenotype. This is consistent with the requirement of the arginase activity for the production of L-ornithine and polyamines for cell proliferation (46).

Moreover, OAT-1746 treatment affects the expression of genes involved in leukocyte chemotaxis (*Ccl2*, *Ccl7*, *Ccl17)* and supporting glioma migration and invasion (*Cme1*, *S100a4* and *Mmp14)*. Ccl2 and Ccl7 control the recruitment of monocytes and neutrophils to the inflamed or tumor tissues, while Ccl17 attracts regulatory T cells (47). MMP14 (MT1MMP) is a metalloproteinase upregulated in GAMs, which activates MMP2 and ECM degradation (48). *Clec12a* and *Clec4a1*, which encode dendritic cell inhibitory receptors, were downregulated in the OAT-1746 group.

Ccl2 is released by many cells present in the tumor microenvironment, including stromal cells, leukocytes, endothelial cells, and malignant cells, which results in augmentation of the plasma chemokine levels (58). Despite decreased expression of *Ccl2* mRNA in CD11b^+^ cells immunosorted from the tumor-bearing brains of OAT-1746-treated animals, the other cells in the tumor microenvironment could still be the source of the cytokine and augment Ccl2 plasma levels. In humans and in animal glioma models, increased CCL2 expression has been associated with high number of GAMs infiltrating tumor tissues, increased angiogenesis and tumor invasion, and poor clinical prognosis (59–61). Impact of increased Ccl2 and decreased Ccl5 plasma levels in OAT-1746 treated animals on the outcome of potential treatment requires further investigation. Measuring the levels of cytokines in the brain or tumor tissue would provide additional insights into the mechanism of action of mono- and combined therapies

Interestingly, the transcriptomic analysis showed the upregulation of several genes implicated in antitumor immune responses such as *Nos2*, *Ifng*, *Tlr4* and *CD86* upon the combinatorial therapy. Genes associated with differentiation and cytotoxic activity of NK cells were significantly upregulated upon anti-PD-1 treatment and COMB therapy. The CD11b^+^ population encompasses NK cells. The upregulation of genes encoding NK surface proteins (*Klrb1f*, *Klre1*, *Klrc2*), cytotoxic granule protein (*Nkg7*), T-Box transcription factor 21 (*Tbx21*), granzyme A (*Gzma*) and perforin (*Prf1*), along with the upregulation of *Serpinb6b* and *Serpinb9b* that protect leukocytes from the granzyme-mediated cell death, suggests the restoration of NK cell functions. Downregulation of genes encoding proteins acting as the suppressors of the immune responses: ApoE, CD69 (50), and Cxcr4 in OAT-1746 and COMB groups indicates the restoration of antitumor functions of CD11b^+^ cells, which may explain the antitumor effect of the combination therapy. These results indicate how important for the effective immunotherapy is the reprogramming of TME. Altogether, our results demonstrate that combining OAT-1746 with PD-1 inhibition may be a promising strategy for the therapy of GBM patients. The complexity of interactions in the tumor microenvironment, arginase inhibition in different cells and anti-PD-1 inhibition resulting in changes of the immune compartment may explain a low number of differentially expressed genes in the COMB group and a small overlap of OAT-1746 mono and combined treatment Future testing of the drug efficacy on established tumors, alternatively to the currently studied preventive treatment regimen, would provide additional information on a mode of action and a translational potential of OAT-1746. ARG1 expression is substantially elevated in myeloid cells in cancer and mitigates antitumor responses *via* multiple mechanisms. Arginase production by macrophages not only leads to the inhibition of antitumor response *via* L-arginine degradation, but also increases the proliferation of tumor cells, which is associated with the production of L-ornithine and then polyamines. Moreover, L-arginine depletion in the tumor microenvironment attenuates nitric oxide (NO) production and reduces its cytotoxic effects on tumor cells (62).

Cytotoxic lymphocytes require exogenous arginine for proliferation (19, 21, 63) and low plasma arginine levels are linked to immunosuppression in cancer patients (64). Arginase 1 expression in malignant cells and myeloid cells in the TME represents a powerful mechanism for tumor immune evasion (65). Dietary supplementation with L-arginine altered the spectrum of TILs and enhanced cytotoxicity in human colorectal and breast cancers (66, 67). Elevation of arginine levels exerted immune-stimulatory effects in various cancers, for example blocking arginase activity with nor-NOHA in leukemic cells induced cell death (68) and treatment with another inhibitor CB-1158 had antitumor effects in several non-CNS cancers in mice (24). Here we demonstrate that a novel, oral ARG1/2 inhibitor, which increases L-arginine levels in the brain and restores the functionality of GAMs and NK cells, sensitizes murine gliomas to the PD-1 inhibition. The combination of OAT-1746 and anti-PD1 leads to the elevation of a number of CD3^+^ T cells in the majority of tumors. Our results support a rationale of combining compounds targeting TME (such as OAT-1746) with PD-1 inhibition as a potential strategy to treat GBM patients.

## Data Availability Statement

The original contributions presented in the study are publicly available. This data can be found here: National Center for Biotechnology Information (NCBI) Gene Expression Omnibus under accession number GSE173865.

## Ethics Statement

The animal study was reviewed and approved by The First Warsaw Local Ethics Committee for Animal Experimentation (approval no 562/2018).

## Author Contributions

BK, PP, and AE-M designed the experiments, evaluated the data, and wrote the manuscript. PP, KW, SC, AE-M, and NO performed the experiments, data interpretation and wrote the manuscript. BG performed RNA-seq and A-JR performed computational analyses, data interpretation and edited the manuscript. MG, PS, RB, and PD synthesized the ARG1/2 inhibitors, performed enzyme inhibition tests and determination of the inhibitor and arginine concentrations, contributed to the data interpretation and edited the manuscript. All authors contributed to the article and approved the submitted version.

## Funding

Studies were supported by the project DIMUNO “Development of new cancer therapies based on selective antitumor immunomodulators”- co-financed by the National Centre for Research and Development, Poland and the Foundation for Polish Science TEAM-TECH Core Facility project “NGS platform for comprehensive diagnostics and personalized therapy in neuro-oncology” (KW, AJ-R, BG, BK).

## Conflict of Interest

MG, PS, and RB are employees of OncoArendi Therapeutics. PD is a former employee of OncoArendi Therapeutics.

The remaining authors declare that the research was conducted in the absence of any commercial or financial relationships that could be construed as a potential conflict of interest.

## Publisher’s Note

All claims expressed in this article are solely those of the authors and do not necessarily represent those of their affiliated organizations, or those of the publisher, the editors and the reviewers. Any product that may be evaluated in this article, or claim that may be made by its manufacturer, is not guaranteed or endorsed by the publisher.
