# Supplementary material for: A Novel Oral Arginase 1/2 Inhibitor Enhances the Antitumor Effect of PD-1 Inhibition in Murine Experimental Gliomas by Altering the Immunosuppressive Environment
Source: Front Oncol. 2021 Aug 24;11:703465. doi: 10.3389/fonc.2021.703465 (PMC8422859; doi:10.3389/fonc.2021.703465)
Supplement: Supplementary file 1 [file DataSheet_1.docx]

Supplementary Material

# Supplementary Figures and Tables

## Supplementary Figures


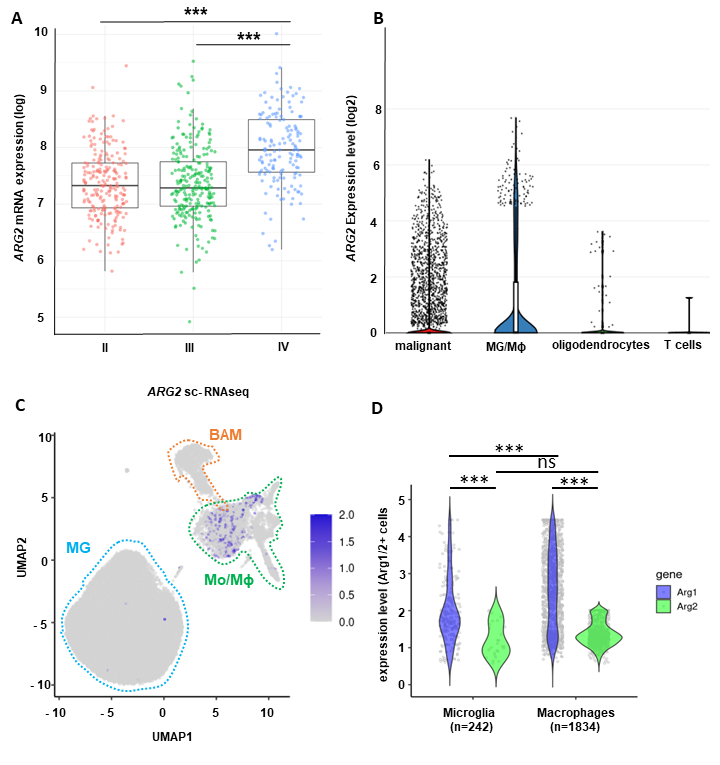


**Supplementary Figure 1. *ARG2* expression is highly upregulated in human glioblastomas and murine experimental gliomas. (A)** *ARG2* expression in gliomas of different WHO grades (WHO grades II- IV) in TCGA datasets. Statistical significance was determined by Tukey’s Honest Significant Difference (HSD); ***p<0.001. **(B)** Expression of *ARG2* in malignant cells and microglia/macrophages (MG/ MΦ) in 10 tumor samples of astrocytomas in single-cell RNA-seq datasets (public data from Ref. 33). **(C)** UMAP plot of CD11b^+^ cells from GL261 gliomas (n=8). Projection of cells combined from clusters identified as microglia, monocytes/macrophages (Mo/MΦ), and BAMs (CNS-border associated macrophages). Plots depict *Arg2* mRNA which is highly expressed in infiltrating Mo/MΦ. **(D)** Violin plots show *Arg1* and *Arg2* mRNA levels in microglia and monocyte/macrophages in the tumor-bearing mouse brain. Data collected for all cells expressing *Arg1* and/or *Arg2* detected by single cell RNAseq. Statistical significance was determined by Tukey’s Honest Significant Difference (HSD). ***p<0.001; ns – not significant.


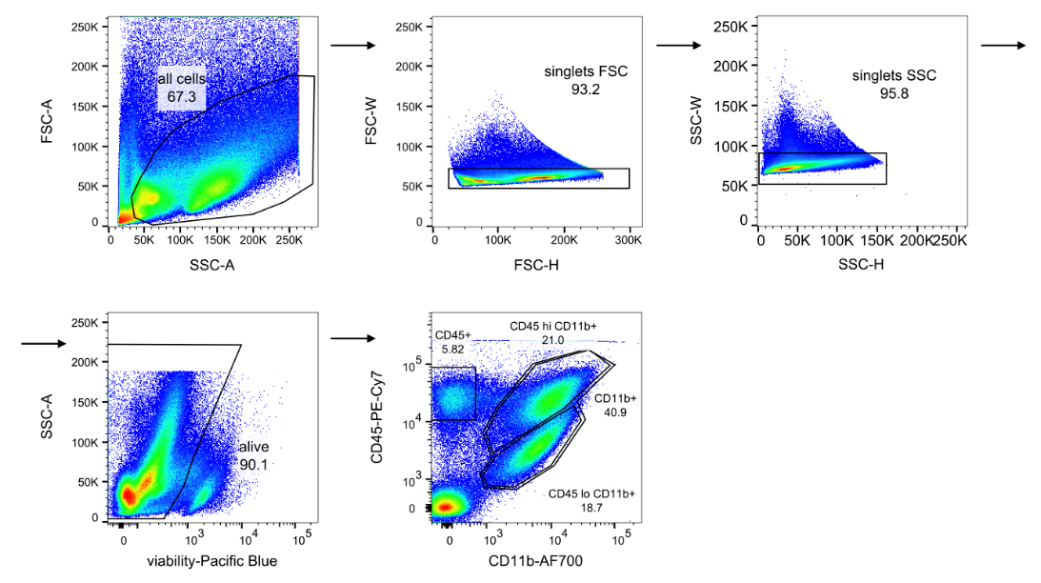


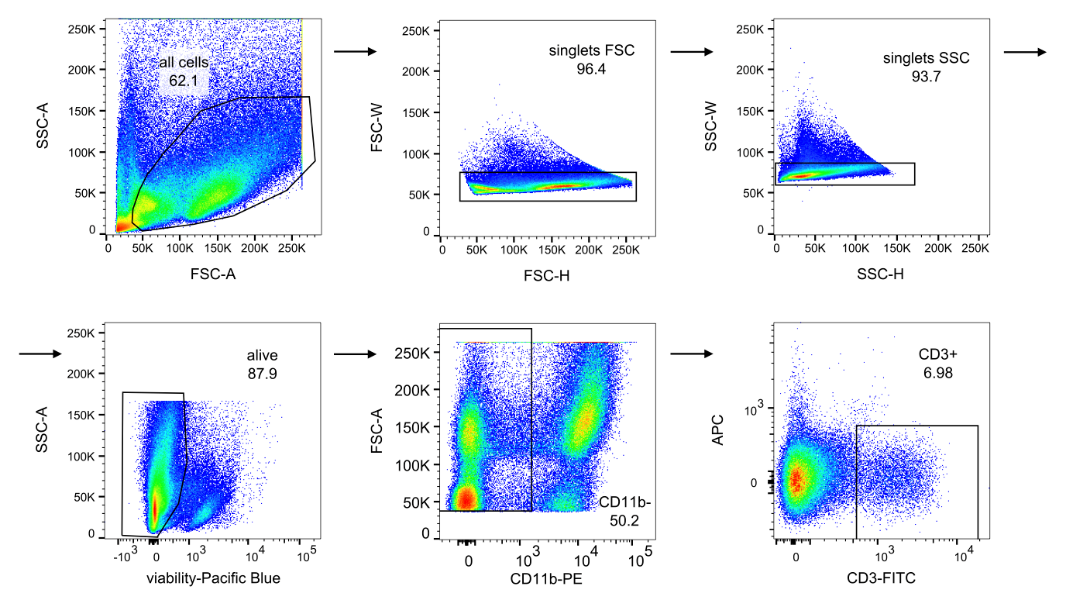


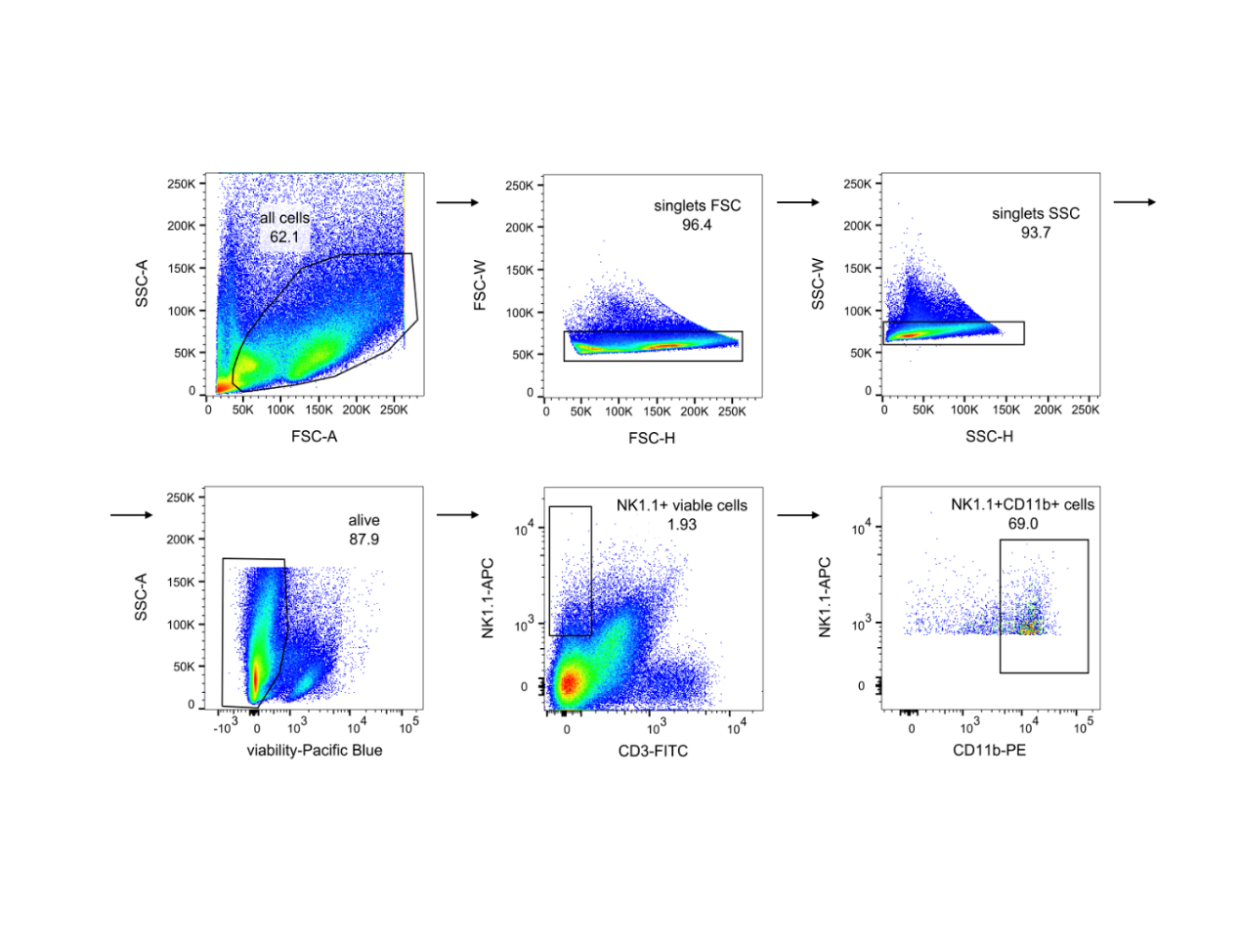


**Supplementary Figure 2.** **Gating strategy.** FSC-W/FSC-H plots were used to determine singlet gates. FSC-A/SSC-A plots were used to determine alive cells. Quadrant gates were drawn on cell

subpopulations based on differences in the surface expression of CD11b and CD45 antigens: microglia (CD11b^+^CD45^lo^), blood-derived macrophages (CD11b^+^CD45^high^ ), CD3 and NK cells.


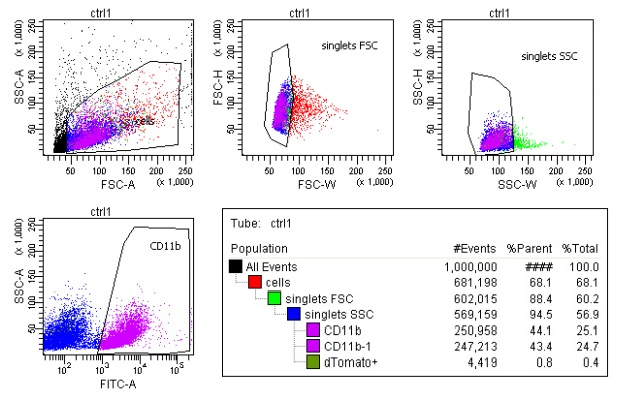


**Supplementary Figure 3.** **CD11b^+^ sorting strategy.** Representative gating strategy for control sample. Quadrant gates were drawn on cell subpopulation based on expression of CD11b labeled with FITC.

## Supplementary Tables

**Supplementary Table 1.** Specifications, catalog numbers and dilutions of reagents used for flow cytometry and immunohistochemistry

| **Reagent** | **Manufacturer** | **Cat. number** | **Clone** | **Fluorophore** | **Application** | **Dilution** | **Lot number** |
| --- | --- | --- | --- | --- | --- | --- | --- |
| Live Dead Fixable Violet Dead Cell | ThermoFisher | L34955 | - | - | FC | 1:1000 | 1910200 |
| Stain Buffer | BD Pharmingen | 554656 | - | - | FC | - | 9329560 |
| Anti-mouse CD16/CD32  Fc Block | BD Pharmingen | 553142 | - | - | FC | 1:250 | 8130843 |
| anti-CD45 mAb | BD Pharmingen | 561868 | 30-F11 | PE-Cy7 | FC | 1:800 | 8205729 |
| anti-CD11b mb | BD Pharmingen | 557960 | M1/70 | Alexa Fluor 700 | FC | 1:800 | 7180930 |
| anti-CD11b mb | BD Pharmingen | 553310 | M1/70 | FITC | FC | 1:800 | 8295813 |
| Anti-NK1.1 mb | Miltenyi Biotec | 130-117-528 | PK136 | APC | FC | 1:200 | 5180716359 |
| anti-CD3 | Miltenyi Biotec | 130-119-758 | REA641 | FITC | FC | 1:10 | 5190917142 |
| anti-Arginase pAb | Novus Biologicals | NB100-59740 | - | - | IF | 1:100 | S262 |
| anti-CD8 alpha | Abcam | ab217344 |  |  | IF | 1:200 | GR3216584-5 |
| anti-Iba1 | Wako | 019-19741 | - | - | IF | 1:1000 | CAF6806 |
| anti-rabbit Alexa Fluor 488 | Invitrogen | A21206 | - | Alexa Fluor 488 | IF | 1:1000 | 1874771 |
| anti-rabbit Alexa Fluor 647 | Invitrogen | A31573 | - | Alexa Fluor 647 | IF | 1:1000 | 1964354 |
| anti-goat Alexa Fluor 488 | Invitrogen | A11055 | - | Alexa Fluor 488 | IF | 1:1000 | 1915848 |
